# Correction: Predicting small molecules solubility on endpoint devices using deep ensemble neural networks

**DOI:** 10.1039/d4dd90020k

**Published:** 2024-05-03

**Authors:** Mayk Caldas Ramos, Andrew D. White

**Affiliations:** a Department of Chemical Engineering, University of Rochester Rochester NY 14627 andrew.white@rochester.edu

## Abstract

Correction for ‘Predicting small molecules solubility on endpoint devices using deep ensemble neural networks’ by Mayk Caldas Ramos and Andrew D. White, *Digital Discovery*, 2024, **3**, 786–795, https://doi.org/10.1039/D3DD00217A.

The header row in Table 2 is incorrect. The correct version of Table 2 is displayed below. Please note that the references are reproduced here as ref. 1–13.

**Table:** Metrics for the best models found in the current study (upper section) and for other state-of-the-art models available in the literature (lower section). Values were taken from the cited references. Missing values stand for entries that the cited authors did not study. SolChal columns stand for the solubility challenges. 2_1 represents the tight dataset (set-1), while 2_2 represents the loose dataset (set-2) as described in the original paper (see ref. 1). The best-performing metrics value are displayed in bold

Model	SolChal1		SolChal2_1		SolChal2_2		ESOL	
	RMSE	MAE	RMSE	MAE	RMSE	MAE	RMSE	MAE
RF	1.121	0.914	**0.950**	**0.727**	**1.205**	**1.002**		
DNN	1.540	1.214	1.315	1.035	1.879	1.381		
DNN_Aug_	1.261	1.007	1.371	1.085	2.189	1.710		
kde4^LSTM^_Aug_	1.273	0.984	1.137	0.932	1.511	1.128	1.397	1.131
kde8^LSTM^_Aug_	1.247	0.984	1.044	0.846	1.418	1.118	1.676	1.339
kde10^LSTM^_Aug_	**1.095**	**0.843**	0.983	0.793	1.263	1.051	**1.316**	**1.089**
Linear regression^2^							0.75	
UG-RNN^3^	0.90	0.74						
RF w/CDF descriptors^4^	0.93							
RF w/Morgan fingerprints^5^		**0.64**						
Consensus^6^	**0.91**							
GNN^7^	∼1.10		**0.91**		**1.17**			
SolvBert^8^	0.925							
SolTranNet^a^^,^^9^			1.004		1.295		2.99	
SMILES-BERT^b^^,^^10^							0.47	
MolBERT^b^^,^^11^							0.531	
RT^b^^,^^12^							0.73	
MolFormer^b^^,^^13^							**0.278**	

a Has overlap between training and test sets.

b Pre-trained model was fine-tuned on ESOL.

The Royal Society of Chemistry apologises for these errors and any consequent inconvenience to authors and readers.

## Supplementary Material

## References

[cit1] Llinas A., Avdeef A. (2019). Solubility Challenge revisited after ten years, with multilab shake-flask data, using tight (SD 0.17 log) and loose (SD 0.62 log) test sets. J. Chem. Inf. Model..

[cit2] Delaney J. S. (2004). ESOL: estimating aqueous solubility directly from molecular structure. J. Chem. Inf. Comput. Sci..

[cit3] Lusci A., Pollastri G., Baldi P. (2013). Deep architectures and deep learning in chemoinformatics: the prediction of aqueous solubility for drug-like molecules. J. Chem. Inf. Model..

[cit4] McDonagh J. L., Nath N., De Ferrari L., van Mourik T., Mitchell J. B. O. (2014). Uniting cheminformatics and chemical theory to predict the intrinsic aqueous solubility of crystalline druglike molecules. J. Chem. Inf. Model..

[cit5] Tayyebi A., Alshami A. S., Rabiei Z., Yu X., Ismail N., Talukder M. J., Power J. (2023). Prediction of organic compound aqueous solubility using machine learning: a comparison study of descriptor-based and fingerprints-based models. J. Cheminf..

[cit6] Boobier S., Osbourn A., Mitchell J. B. O. (2017). Can human experts predict solubility better than computers?. J. Cheminf..

[cit7] Panapitiya G., Girard M., Hollas A., Sepulveda J., Murugesan V., Wang W., Saldanha E. (2022). Evaluation of Deep Learning Architectures for Aqueous Solubility Prediction. ACS Omega.

[cit8] Yu J., Zhang C., Cheng Y., Yang Y.-F., She Y.-B., Liu F., Su W., Su A. (2023). SolvBERT for solvation free energy and solubility prediction: a demonstration of an NLP model for predicting the properties of molecular complexes. Digital Discovery.

[cit9] Francoeur P. G., Koes D. R. (2021). SolTranNet–A Machine Learning Tool for Fast Aqueous Solubility Prediction. J. Chem. Inf. Model..

[cit10] Kim H., Lee J., Ahn S., Lee J. R. (2021). A merged molecular representation learning for molecular properties prediction with a web-based service. Sci. Rep..

[cit11] Fabian B. , Edlich T., Gaspar H., Segler M., Meyers J., Fiscato M. and Ahmed M., Molecular representation learning with language models and domain-relevant auxiliary tasks, arXiv, 2020, preprint, arXiv:2011.13230, 10.48550/arXiv.2011.13230

[cit12] Born J., Manica M. (2023). Regression Transformer enables concurrent sequence regression and generation for molecular language modelling. Nat. Mach. Intell..

[cit13] Ross J., Belgodere B., Chenthamarakshan V., Padhi I., Mroueh Y., Das P. (2022). Large-scale chemical language representations capture molecular structure and properties. Nat. Mach. Intell..

